# Development of a Sensor Node for Precision Horticulture

**DOI:** 10.3390/s90503240

**Published:** 2009-04-28

**Authors:** Juan A. López, Fulgencio Soto, Pedro Sánchez, Andrés Iborra, Juan Suardiaz, Juan A. Vera

**Affiliations:** 1 DSIE, Technical University of Cartagena, Campus Muralla del Mar s/n, Cartagena, E-30202 Spain; E-Mails: p.soto@upct.es (F.S.); pedro.sanchez@upct.es (P.S.); andres.iborra@upct.es (A.I.); juan.suardiaz@upct.es (J.S.); 2 Edosoft Factory S.L., Antonio María Manrique 3, Las Palmas de Gran Canaria, E-35011 Spain; E-Mails: juan.vera@edosoftfactory.com (J.V.)

**Keywords:** Wireless Sensor Networks, Mote, TinyOS, Precision Horticulture

## Abstract

This paper presents the design of a new wireless sensor node (GAIA Soil-Mote) for precision horticulture applications which permits the use of precision agricultural instruments based on the SDI-12 standard. Wireless communication is achieved with a transceiver compliant with the IEEE 802.15.4 standard. The GAIA Soil-Mote software implementation is based on TinyOS. A two-phase methodology was devised to validate the design of this sensor node. The first phase consisted of laboratory validation of the proposed hardware and software solution, including a study on power consumption and autonomy. The second phase consisted of implementing a monitoring application in a real broccoli (*Brassica oleracea* L. var Marathon) crop in Campo de Cartagena in south-east Spain. In this way the sensor node was validated in real operating conditions. This type of application was chosen because there is a large potential market for it in the farming sector, especially for the development of precision agriculture applications.

## 1. Introduction

One means to achieve enhanced efficiency in crop farming and water resources is Precision Agriculture [1], for which WSN (Wireless Sensor Networks) is one promising technology [2]. In fact, a number of studies have been published in the last few years on applications of this kind using WSN in precision agriculture. For example, Camilli *et al.* [3] used a simulation to demonstrate the utility of a WSN in this application; Pierce *et al.* [4] described a platform that they had developed for local and regional networks and implementations of them in two precision agriculture applications in Washington State; and Morais *et al.* [5] described a prototype that they had developed for deployment in vineyards to monitor crops of this type.

At the same time, the interest aroused by this technology has prompted the appearance on the market of various hardware platforms (sensor nodes or motes) for the development of new applications (hospitals [6], unfriendly environments [7], mobile control applications [8], etc.) and research work (energy-efficient optimization [9], target tracking [10], etc.). Worth noting among these platforms are MICAz™, TelosB™, IRIS™, Imote2™ and others. Ot should be noted that these motes, and others not mentioned here, are devices that normally include embedded low-cost sensors whose specifications (precision, resolution, drift, etc.) are not the same as those of the instruments that are normally required in Precision Agriculture applications. For instance, the TelosB™ that includes the temperature sensor Sensirion SHT11 has no protection level. Moreover the temperature accuracy of this sensor is very dependent on the environmental conditions compared to the HMP41 Vaisala™ sensor, widely used in precision agriculture. Also, many of these motes have only been used in laboratory or research applications and are not robust enough for use in real agricultural environments. In this regard, some years ago Crossbow [11] began to market the MEP-SYS kit for monitoring environmental parameters. This product, comprising two types of nodes, can be used to monitor ambient temperature, relative humidity, radiation and barometric pressure. Moreover, thanks to their protective casing these motes can be used for both open-air and greenhouse crops. The same manufacturer recently marketed the eKo kit, which offers greatly improved robustness as compared to the previous product. It further incorporates a small solar panel, and most importantly, it can be linked to up to four sensors, either Crossbow or other makes, by 2- or 3-wire interfaces. Some other manufacturers are proposing similar solutions to Crossbow's [12,13]. In other hand, there are others platforms developed in the framework of research projects [14,15] that uses general purpose motes (MICAz™, TelosB™, etc) enclosed inside watertight boxes.

Another important aspect of the motes is their capacity for connection with external instruments. There is a large group of sensors used in the field of precision agriculture such as the Hydra Probe II and the EC 1200 of Stevens Water Monitoring Systems, Inc which provide output by means of the SDI-12 protocol [16]. Another major distributor, Campbell Scientific, markets sensors like the CS245-L, CS408-L and WINDSONIC4-L. Consequently, if motes are to be used in real agricultural applications, they first of all need to incorporate the necessary electronics to connect with external quality instruments. For it, one of our primordial objectives has been to see that our motes can connect directly to this type of SDI-12 sensors. This feature together with robustness, autonomy and the possibility of connecting different types of instruments have been prioritized, as opposed to other functional characteristics less primordial, such as, wireless reprogramming, and the implementation of complex algorithms in the motes. It is important to mention that has not been found in the scientific literature any reference of a mote that provides a SDI-12 interface.

This paper describes a sensor node (GAIA Soil-Mote) with a SDI-12 interface and IP67 protection level. The mote allows for the installation of a large number of agricultural sensors in a crop, with wireless data transfer to a centrally-located base station. This study provides the reader with the main key characteristics of the developed sensor node.

After this introduction, Section 2 gives a detailed description, in terms of hardware and software, of the device developed for implementation of wireless sensor networks for precision horticulture. Section 3 describes the two experimental scenarios in which the sensor node was tested. The first one was a network scattered in a laboratory with a smaller number of nodes than the second, deployed in a real crop. This is the main experimental part of the paper, and the results obtained confirm that the hardware and software solutions proposed do indeed lead to good performance. The conclusions and future plans are discussed in Section 4, which closes the paper.

## 2. Development of a Wireless Sensor Mote for Precision Horticulture

When choosing design and construction requirements for a sensor node, it may be necessary to make a compromise between conflicting requirements. In the design of the GAIA-Soil-Mote, priority was awarded essentially to architectural decisions that favoured system robustness and also interconnection of different types of sensors. It was only once these premises and the requisite compatibility for communications had been established that additional requirements to render the sensor node more suitable and functional were considered. The new mote was developed with the following goals in mind:

1. The mote must be able to communicate with other motes via a highly reliable radio module compatible with the IEEE 802.15.4 standard [17].
2. It is proposed to develop a robust product to monitor soil parameters (temperature, humidity, salinity and electrical conductivity) in crops that may be distributed in plots averaging 4 ha in size and situated up to 10 km apart.
3. The mote must have a SDI-12 interface so that any sensor using this protocol can be connected.
4. The mechanical design of the device must be optimized for use on horticultural crops. This means that it must be suitable for installation at ground level and be small enough not to have to be removed when the crop is fumigated using farm machinery. The mote's casing must therefore provide IP67 standard protection and must be no more than 20 cm high (including the antenna). Also, to help avoid the motes being stolen, they should be painted a discreet colour. Note that although the design must be optimized for horticultural crops, the motes must also be able to be used in other kinds of crops such as fruit crops, vineyards, etc.
5. They must be battery-operated, with an autonomy of at least 10 weeks, which is the normal duration of a horticultural crop cycle.
6. Applications with the mote must be simple to develop with the aid of standard programming and debugging tools.
7. The mote must have a single external connector. This connector will serve for programming and debugging the node software, resetting it, charging the battery and connecting to the external sensors.
8. The sensor node must be susceptible of wireless configuration for reading of the sensors with a frequency ranging from 30 minutes to 10 days. Also, the node will send the battery level to the monitoring software every hour.

The following sections describe the hardware and software used in the sensor node that has been developed. It also includes a subsection with information on the SDI-12 external sensors used to measure the soil characteristics.

### 2.1. Description of the Hardware

**Figure 1.**
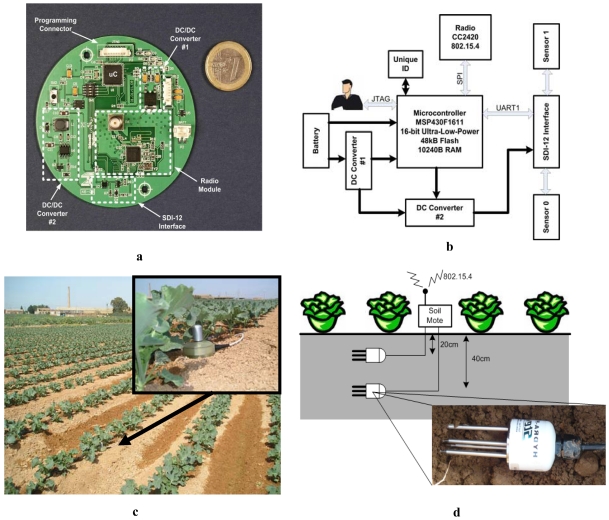
Different views of the GAIA Soil-Mote. (a) PCB. (b) Block diagram. (c) External view of casing. (d) Image of device in field with detail of the sensor used.

Figure 1 shows the high level of integration of the sensor node that has been developed. The PCB (Figure 1a) is round in shape, with a radius of 4 cm. Figure 1b shows a block diagram of the mote. The mote card has been developed with a minimal number of components. This is due in part to the low power-consumption requirement and in part to the need to keep the mote size and manufacturing costs to a minimum. The core of the platform is a MSP430F1611 ultra-low power microcontroller from Texas Instruments. This architecture, combined with five low-power modes, is optimized to achieve extended battery life in portable measurement applications. The device is a powerful 16-bit RISC CPU with constant generators to achieve maximum code efficiency. The digitally controlled oscillator (DCO) allows wake-up from low-power modes to active mode in less than 6 μs. This micro can typically operate at clock frequencies up to 8 MHz and address up to 10 KB of memory (much less is used in practice). This module is contained in a 9.1 mm × 9.1 mm QFN package. Wireless communication is provided by the Chipcon CC2420 radio module. This circuit combines low power and efficient operation with support for IEEE 802.15.4. It operates in the 2.4 GHz Industrial-Scientific-Medical (ISM) free radio frequency band, with 16 channels. The radio module is connected to a 2 dBi omnidirectional antenna for outside use. An “SDI-12 Interface” module has been included to enable the external sensors to be connected to the microcontroller's UART. This interface is necessary for two reasons: the microcontroller and the external sensors work with different voltages, and moreover the two pins of the UART (Rx and Tx) have to be interconnected with the single two-way data line from the external sensors. All this is achieved by means of a tri-state buffer, a transistor and some discrete components so as to incorporate the SDI-12 interface in the mote. A single connector serves to connect up to 10 external sensors. Note that using only one external connector simplifies the PCB design of the board.

The mote is powered with the help of a 3.7 V, 2,000 mAh LiPo lithium polymer battery. When the battery is fully charged it delivers more than 4 V. For that reason, as the block diagram shows, a low-consumption, low-dropout DC/DC converter (#1) is included to keep the battery voltage at 2.5 V. This voltage powers the microcontroller and the radio module. Then there is a second converter (#2) which transforms the 2.5 V to provide the 12 V output required by the external sensors. For efficient energy management, this converter is enabled only during the sensor reading process. Finally, the block diagram shows the battery connected to the microcontroller by a voltage divider to allow periodic sampling of the battery level.

The mote includes a basic user interface using three LEDs and one 12-pin external connector. The LEDs are used to show the state of the mote (after reset, sending a message, etc.) and the external connector has the following features: mote reset, battery charge, software programming and debugging using JTAG, and connection to the external sensors.

Figure 1c shows the deployment of the GAIA Soil-Mote in the experimental crop. The assembly is enclosed in a casing which provides IP67 standard protection. It is suitable for this type of environments with dust and water. Figure 1d shows the location of the mote in a horticultural crop. The sensor node is connected to two Stevens Hydra Probe II (HP2) sensors, which are described further below. The HP2 sensors are buried at different depths to monitor the main soil parameters.

### 2.2. Description of the Software

The device software was developed with TinyOS version 2.0 [18] as an operating system environment with the nesC component-oriented programming language associated [19]. TinyOS is an event-driven open-code operating system that provides the functionality needed for the proposed application, and it benefits from a large and active user community. We ported TinyOS to our platform and used a set of drivers that make the various I/O components available to the application programmer.

**Figure 2.**
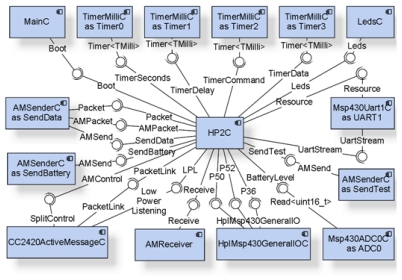
GAIA Soil-Mote: nesC component diagram.

Figure 2 shows the nesC component diagram for the mote as developed. HP2C is a new component that is grafted on to TinyOS and is optimized for the use of two HP2 sensors. All the other components are operating system primitives which have been instantiated (referenced in the blocks of the component diagram as “*Name_of_Predefined_Component* a*s Name_of_Instantiated_Component*”.

The program entry point is supplied by the MainC component using the Boot interface. For the program to function, it is necessary to instantiate four TimerMilliC components, which are required to manage the program timer so as to know when to take a reading (Timer 0), send the command to start up the reading process (Timer 2) and start the data reading process (Timer 3), and to be able to handle a time lag from when the process starts until the data are available (Timer 1).

For communications, three AMSenderC components are required, one of the type CC2420ActiveMessageC and another of the type AMReceiver. The SendData instance of the AMSenderC component is used to send the data gathered from the sensors to the sink node. The SendBattery instance sends the battery level data every hour. Finally, to facilitate the process of node deployment with the SendTest instance, a test message received from the Base Station is echoed. With the AMReceiver component it is possible to receive two types of messages: test messages and messages to alter the sensor reading timing. The last component, CC2420ActiveMessageC, is used to initiate the radio module, set the number of retries and the delay between them, and manage its operating time. In this way energy expenditure by the radio transceiver can be optimized. The nodes are specifically configured with a low-power-listening (LPL) period of 10 s. On this point it is important to remember that the frames sent and received with the selected TinyOS components are compatible with standard IEEE 802.15.4. We say that “*the frames are compatible with the standard*” because in order to optimize consumption TinyOS uses the BMAC environment access protocol instead of the MAC protocol, which is the one actually defined in the standard.

The Msp430Uart1C component is the one used to manage all communications with the HP2 sensor. With the LedsC component it is possible to access the 3 LEDs included in the mote, which are used to indicate states such as sending data from the mote, receiving a message and others. Msp430ADC0C allows sampling of the microcontroller ADC0 that is connected to the battery by way of a resistive voltage divider. Finally, a HPL (Hardware Presentation Layer)-level TinyOS component is needed to manage a series of microcontroller pins required for management of the SDI-12 interface (communications and DC/DC converter on/off).

### 2.3. Hydra Probe II sensor

The Hydra Probe II sensor (HP2) (see Figure 1d) is a commercial product from Stevens Water Monitoring Systems, Inc. which is available with two different output interfaces: SDI-12 and RS485. Of these two versions, the most suitable for applications with low-consumption requirements is the SDI-12, since consumption in standby mode is 10 times less than with the RS485. Moreover, it only requires three wires (power, earth and data) as opposed to the four required by the RS485 (power, earth, com+ and com-). This sensor can provide various parameters for the ground, the most important being the temperature, volumetric percentage, conductivity and salinity of the soil. Calibration is carried out in the sensor itself, so that the data are received in digital-format physical units and ASCII code. Nevertheless, users may wish to perform their own calibration, and there is therefore the possibility of receiving uncalibrated data on the variables directly from the ADCs, in raw format. Communication is achieved by sending ASCII commands and interpreting the response from previous ones, as in any SDI-12 device. These commands can be used to take readings of the above-mentioned values and to configure parameters such as soil type, water constant, setting-up time and definition of the variables to be measured.

## 3. Experimental Results

To assess the proper functioning of the GAIA Soil-Mote, a methodology was devised in two experimental phases: laboratory tests and trials on a real crop. The main function of the first phase was to validate the proposed hardware architecture and software solutions. The hardware was validated with the software versions that did not use low-consumption techniques. Once proper functioning was assured, the next step was to review the software in order to incorporate low-consumption modes in all the devices except those powered from the grid. In this way we achieved sufficient autonomy to ensure that the motes would function throughout a horticultural cycle (10 weeks). The object of the second phase, which was conducted in real conditions on the farm, was to assess the functional performance of the devices that had been developed, such as range, robustness and flexibility.

### 3.1. Laboratory experiments

**Figure 3.**
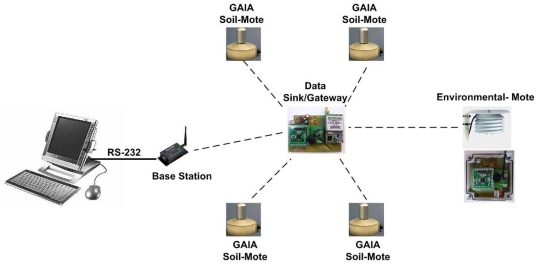
The system architecture for the laboratory experiment.

In the first phase, a network was deployed similar to the one that would eventually be deployed on the real crop, but with a smaller number of nodes (see Figure 3). The network was composed of four GAIA Soil-Mote to monitor the state of a crop's soil. A new node (Environmental-Mote) was also added to monitor the crop's ambient temperature and humidity, again with the help of an external sensor (Sensirion SHT71). The architecture of this last sensor node differed from that of the GAIA Soil-Mote in its interface module. In this case connection with the external sensor was achieved via the I2C interface. Figure 3 shows that the sensor data are transmitted directly from the sensor node to the Data-Sink/Gateway, which then transmits them to the Base Station. The Data-Sink/Gateway architecture is very similar to that of the GAIA Soil-Mote, as in the case of the Environmental-Mote,. In this case, an XStream radio modem was connected to the microcontroller by way of the UART. Ranges of up to 10 km have been confirmed with this device.

**Figure 4.**
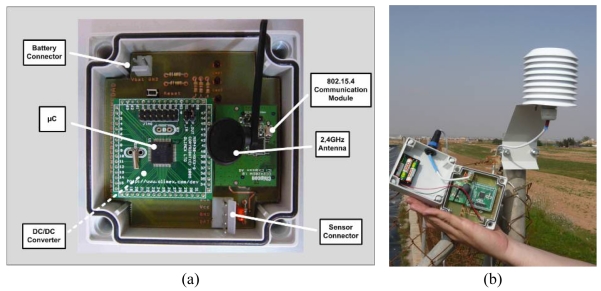

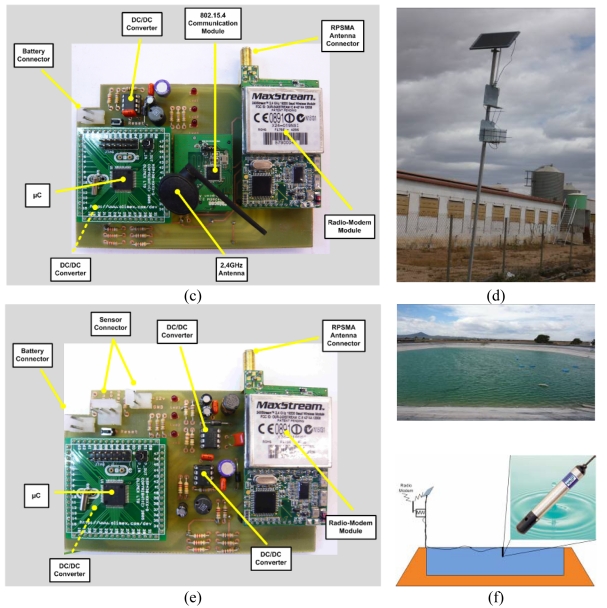
Views of the different prototypes developed with similar architecture to the GAIA Soil-Mote. (a) Environmental-Mote. (b) Environmental-Mote installed in the field. (c) Data-Sink/Gateway. (d) Data-Sink/Gateway installed in the field. (e) Water-Mote. (f) Water-Mote installed in the field.

Table 1 summarizes the main characteristics of each of these devices. Both the Environmental-Mote and the Data-Sink/Gateway were available in prototype form, implemented with inserted electronic components (microcontroller, radio transceiver, DC/DC converters, passive components, etc.). All the devices are battery-powered except for the Base Station, which is powered directly from the grid. Note also that the Data-Sink/Gateway incorporates a solar panel to recharge the 12 V and 6.5 Ah battery. The sensor nodes are not equipped with the solar panels because they need to be camouflaged within the crop to avoid vandalism. Also, plants would throw shade over the sensor nodes, thus reducing the efficiency of the solar charging system. Figure 4 shows some photographs of the prototypes mentioned and their placement in the field during the second phase of the experiment.

**Figure 5.**
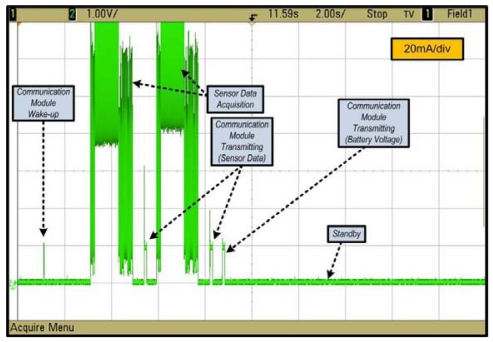
Consumption states of the GAIA Soil-Mote: “standby” = 0.25 mA, “communication module wake-up” = 20 mA, “acquisition” ≈ 110 mA, “communication module transmitting (Sensor Data and Battery Voltage)” = 25 mA.

After functional validation, the devices that had been developed were reprogrammed for low consumption as the mode best suited for operation in the field. A detailed study of this mode of operation was therefore necessary to assure autonomy throughout an agricultural cycle. The study that was carried out for the GAIA Soil-Mote is presented below. The sensor node has four functional states: standby for messages, communication module wake-up, sensor data acquisition and data transmission. Figure 5 shows the mote's power consumption in each of these states.

The worst-case scenario was taken for average consumption, that is acquisition and transmission of data from the two sensors every thirty minutes. The ultimate aim of this study was to determine how much power the mote consumed on average, so as to relate the resulting figure directly to the capacity of the batteries and hence determine the device's autonomy. The mote's average power consumption may be determined thus:

(1) $${\bar{\text{I}}}_{\mathrm{soil-mote}}={\bar{\text{I}}}_{\text{standby}}+{\bar{\text{I}}}_{\text{radio\_wake−up}}+{\bar{\text{I}}}_{\text{sensor\_acquisition}}+{\bar{\text{I}}}_{\text{transmitting}}$$

Where:

(2) $${\bar{\text{I}}}_{\text{standby}}=0.25\text{mA}$$

(3) $${\bar{\text{I}}}_{\text{radio\_wake−up}}\approx \frac{20\text{mA}*15*10^{-3}}{10}=0.03\text{mA}$$

(4) $${\bar{\text{I}}}_{\text{sensor\_acquisition}}\approx 2\cdot \left(\frac{110\text{mA}*1800*10^{-3}}{1800}\right)=0.22\text{mA}$$

(5) $${\bar{\text{I}}}_{\text{transmitting}}\approx 2.5\cdot \left(\frac{25\text{mA}*125*10^{-3}}{1800}\right)=0.00434\text{mA}$$

Expression (2) shows consumption at rest. Equation (3) expresses the average consumption in receiving mode for a 15 ms pulse every 10 s, with the radio switched on in the Low-Power-Listening period. Equations (4) presents a similar calculation, in this case multiplied by two, which is the number of sensors that are connected, and divided by 1,800 s (30 minutes), which is the worst-case scenario for estimating autonomy. Equation (5) expresses the average consumption for the transmission of the sensor data and battery voltage. The equation is multiplied by 2.5 because the messages of the battery voltage are sent every hour. Equation (1) gives us an average current of 0.5043 mA, which, considering that the batteries are 2,000 mAH, gives an estimated autonomy of 165 days. Note that the intensities of each state have been measured at the device input for a voltage of 3 V.

**Figure 6.**
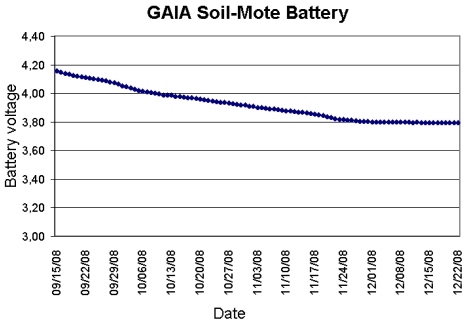
Evolution of battery rundown during laboratory tests.

To check the above result, a real study was conducted in the laboratory (see Figure 6) between September and December 2008, with the device taking sensor readings every 30 minutes. At the end of the study, the final value for the battery was approximately 3.8 V, well above the value of 3.4 V necessary to recharge it. The value required at the main DC/DC converter input for the system to work properly is 2.7 V.

### 3.2. Agricultural results

**Figure 7.**
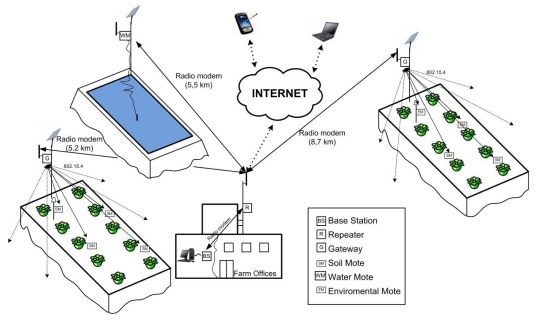
Illustration of the on-going implementation of an in-field data acquisition network, based on the GAIA motes in a precision horticulture environment.

The laboratory tests were followed by the second phase of the experiment, consisting of the deployment shown in Figure 7. Two sensor networks were deployed in two plots of approximately 4 ha each. The plots are about 5.2 and 8.7 km respectively from the farm offices. Ten GAIA Soil-Motes, one Environmental-Mote and one Data-Sink/Gateway were deployed on each of the plots.

The Data-Sink/Gateway was connected to an exterior 3 dBi omnidirectional antenna placed on a post 5 m tall. This was to assure direct line-of-sight between the antennas of the ground-level Motes and the Data-Sink/Gateway. For the same reason, to assure line-of-sight between the Data-Sink/Gateway and the offices, one 15 dBi omnidirectional antenna was placed on the office roof (9 m high), and another identical antenna on the Data-Sink/Gateway. Wireless communication between the rooftop antenna and the Base Station was achieved with a repeater. At the same time, a technology similar to that of the Data-Sink/Gateway was used to develop a wireless node (Water-Mote) to monitor the quality of the water supplied to the crop using the EC250 sensor (see Figure 7).

A monitoring application, running on the computer connected to the Base Station, was developed to control all the devices and keep a record of the information received. It is integrated by: (1) a graphical user interface (GUI) where the data read by the sensors is shown using the utility supplied by Google Maps, and (2) a program that receives and stores data from the nodes. Both programs were developed using the Java programming language, with the Eclipse environment and the MySQL relational data base management system.

**Figure 8.**
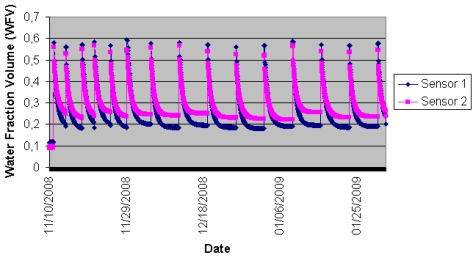
Humidity data from one of the motes.

The trials were conducted on crops of broccoli (*Brassica oleracea* L. var Marathon) covering an area of 4 ha each, located in Campo de Cartagena (37°44′26″N, 1°13′38″W) in south-east Spain. The seedlings were transplanted (with a population density of five plants/m^2^) on 10 November 2008 and the crop was harvested 12 weeks later, in the first week of February 2009. The soil characteristics of the crop at a depth of 40 cm were: clay loam texture, total carbonates 35.4 p.100, P(Olsen) 78.6 ppm, K(Ac-NH4) 487.0 ppm. A drip irrigation system was laid between the two rows of crops and 1 L/h emitters were installed every 0.20 m. Fertilizer was applied to the crop using fertirrigation. The nodes were deployed in the second week of November, at which time the owners of the farm began to gather data from the WSNs. The Soil-Mote sensors were placed at depths of 20 cm and 40 cm. During this time there was 198 mm cumulative rainfall, moderately strong winds of up to 69 km/h and mild temperatures (average 11.5 °C). Figure 8 shows the data (soil moisture) collected over a twelve-week period. The Hydra Probe sensors provide accurate soil moisture measurements in units of water fraction by volume (wfv or m^3^ m^-3^) — that is, a percentage of water in the soil displayed in decimal form. For example, a water content of 0.20 wfv means that a one-litre soil sample contains 200 mL of water. Full saturation (all the soil pore spaces filled with water) typically occurs between 0.5 – 0.6 wfv and is quite soil dependent. The nodes were found to function properly. This provides some assurance of the robustness of the arrangement for similar weather conditions.

The sensor data and battery voltage transmissions were configured with ten and five retries respectively and with a delay of 100 ms for both transmissions. When the limit of ten or five retries is reached, a transmission error is annotated in the motés memory. We checked these values when the field trials finished and no error of this kind was observed. In addition, the autonomy of the devices that had been developed was tested in real operating conditions. The field trials also showed that the ranges specified by the manufacturers were correct. It is important to note here that before developing the GAIA Soil-Mote, we tested different antennas with an earlier prototype. Finally, using the selected antennas we have achieved ranges of up to 100 m between the sensor nodes and the Data-Sink/Gateway in unfavourable environmental conditions, for example when there is high ambient humidity (> 70%). Also, it was verified that the 8.7 and 5.2 km links between the Data-Sink/Gateways and the repeater on the office roof worked properly.

Before this technology was introduced, the company monitored its crops in the traditional way—that is, a person visited the crop and the pond to measure the relevant agronomic parameters with appropriate portable equipment. Now, with the technology that has been developed, crop variables can be ascertained in real-time, and as a result the water requirements of the crops can be estimated without anyone having to visit them. The farm team were able to check, in real-time, that the optimum conditions for broccoli growth were being maintained (salinity in the range 2-4 mmhos/cm, temperature between 10 °C and 24 °C, and relative humidity in the range 60%-90%).

## 5. Conclusions

A sensor node has been designed and manufactured that is robust enough for use in horticultural applications and can measure the following soil parameters: temperature, volumetric percentage, electrical conductivity and salinity. The device has been equipped with a SDI-12 interface to support two Stevens Hydra Probe II sensors. The software has been designed for ready adaptation to any other type of SDI-12 sensors since this interface is provided by a wide range of agronomical sensors. The hardware for the node that has been developed can likewise be adapted to any other type of interface. It has allowed the easy development of two additional nodes with a similar design to the GAIA Soil-Mote: the Environmental-Mote and the Water-Mote. This approach is different from other solutions where the motes are not designed for working in open-air and greenhouse crops.

We are currently working on an improved version of the GAIA Soil-Mote. The aim is to allow industrial manufacture of all the sensor nodes presented in this article, and the Data-Sink/Gateway, using a single PCB. This version will also support more types of output interfaces (SDI-12, 2 – 20 mA, 0 – 2.5 V, I2C).

## Figures and Tables

**Table 1.** Summary of devices used in the laboratory experiment.

**Device**	**Function**	**μC/O.S.**	**Energy Source (Battery Type)**	**Communication Module (Range)**	**Sensors**

GAIA Soil-Mote (final product)	Instantly measures soil moisture, conductivity, salinity, and temperature	MSP430F1611 TinyOS 2	Rechargeable Battery (2000 mAh LiPo lithium polymer)	CC2420 (230 m)	Hydra Probe II (Stevens)
Environmental Mote (Prototype)	Instantly measures relative humidity and temperature	MSP430F1611 TinyOS 2	Rechargeable Battery (3×AA NiMH 2700mAh)	CC2420 (230 m)	SHT71 (Sensirion)
Data Sink/Gateway (Prototype)	Links soil and environmental motes with repeater	MSP430F1611 TinyOS 2	Solar Cell + Rechargeable Battery (12V, 6.5Ah Lead Acid)	CC2420 (230 m) XStream (16 km)	N/A
Base Station (Commercial)	Links WSM with software application	N/A	Grid (N/A)	XStream (16 km)	N/A
